# Global threat of COVID 19 and evacuation of the citizens of different countries

**DOI:** 10.3906/sag-2004-21

**Published:** 2020-04-21

**Authors:** İrfan ŞENCAN, Semanur KUZİ

**Affiliations:** 1 Infectious Diseases and Clinical Microbiology, University of Health Sciences,Dışkapı Yıldırım Beyazıt Teaching and Research Hospital, Ankara Turkey; 2 Infectious Diseases and Clinical Microbiology, Artvin State Hospital, Artvin Turkey

**Keywords:** Quarantine, evacuation

## Abstract

Beginning from China on December 2019, COVID-19 epidemic has spreaded all over the world in a short period of time and has been a pandemic. In challenge with this pandemic quarantine technique has been used widely after tens of years. In the course of the pandemic, many countries evacuated their citizens from affected regions and combined the evacuation with quarantine process. Some examples of these countries who evacuated their citizens are Germany, Italy, Spain, and USA. In further times, during the course of pandemic, according to spread, other countries evacuated their citizens from these countries too. Despite being the origin of the pandemic, in later times Wuhan was also a place where people were evacuated to. Evacuation and quarantine have caused social and psychological impacts on people and some of them took place in mainstream media. In this review article, evacuation and quarantine processes as well as the society’s reactions to these, have been compiled.

## 1. Introduction 

### 1.1. Outbreak of the novel coronavirus

Coronaviruses are a family of viruses which can cause broad spectrum of respiratory tract infections from a simple common cold to more serious respiratory system infections like MERS-CoV (Middle East Respiratory Syndrome) and SARS-CoV (Southern Asia Respiratory Syndrome).

In December 2019, a new coronavirus epidemic, which firstly emerged in China and by 27 March 2020 disseminated almost to all countries and all the regions in the world, was identified. The disease caused by this newly identified coronavirus subtype named as coronavirus 2019 (COVID-19). Eventually International Virus Taxonomy group named this virus as SARS-CoV2 [1].

New coronavirus cases firstly appeared in December 2019 as clusters of pneumonia cases with no identifiable cause in Wuhan city located in China’s Hubei state.

On 30 December 2019, active case finding was initiated in Wuhan and on 31st of December WHO (World Health Organization) was informed about the epidemic^1^1https://www.who.int/docs/default-source/coronaviruse/situation-reports/20200121-sitrep-1-2019-ncov.pdf?sfvrsn=20a99c10_4. On 1 January 2020, Huanuan Seafood Market, where this pandemic arouse from according to the Chinese officials, was closed.

On 6 January 2020, Centre of Disease Control (CDC) announced Level 2 emergency status in China and recommended government to take extra measures in entries to and exits from China.

Level 2: Implementation of Enhanced Precautions^2^2https://wwwnc.cdc.gov/travel/notices: 

1- Elderlies and people who have chronic diseases in all age groups should think of delaying the travel.

2- Travellers should avoid contact with ill people and wash their hands properly at least for 20 s. If there is no possibility to make it happen always, they shall carry and use hand disinfectants.

3-Travellers must watch out their own health status carefully and keep the social distance with others.

4-Travellers who have cough, fever or dyspnoea should stay at home and inform medical staff before getting help.

Virus was isolated from clinical samples on 7 January 2020. On 12 January 2020, China shared new coronavirus’s genetical sequence with the world in order to start developing the diagnostic kits^3^3https://www.who.int/docs/default-source/coronaviruse/who-china-joint-mission-on-covid-19-final-report.pdf. 

### 1.2. Spread of the outbreak all over the world

The first confirmed case outside of China was detected in Thailand. It was an imported case from Wuhan. Thailand Ministry of Public Health took measures at the airports starting from 3 January 2020 in order to scan passengers coming from Wuhan and activated surveillance activities in public and private hospitals.

Starting from 14 January 2020 people who want to leave Wuhan had to undergo detailed medical scanning and it was forbidden them to leave city if they have fever over 37.3 °C. On 15 January, first confirmed case imported from Wuhan was notified in Japan. On 15 January it was announced that China is risky to travel as Level 3 Emergency Responses are determined for the country by CDC^4^4https://wwwnc.cdc.gov/travel/notices/warning/coronavirus-global. It was recommended to avoid travels to/from China.

On 19 January 2020, first case outside of Wuhan city was notified in China. Chinese government sent diagnostic kits to all states in the country. On 20 January 2020 South Korea announced first novel coronavirus case imported from Wuhan, this followed by South Korea announcing national emergency level from blue to yellow. In addition, South Korea increased the efforts to follow all pneumonia cases in the country, established check points throughout the country for quarantine and scanning [2].

On 20 January 2020, COVID 19 was announced as class B notifiable disease. On the same day new coronavirus caused pneumonia was added to Chinese quarantine law^5^5https://www.who.int/docs/default-source/coronaviruse/who-china-joint-mission-on-covid-19-final-report.pdf.

On 23 January 2020, Wuhan and later on 24th, all other 15 cities in Hubei were locked down. Cases in these cities had been handled carefully, treatments as well as protective measures for others were being applied.

On 21 January, the first case in USA was confirmed. Again, in USA on 30 January, the first confirmed case obtained through a transmission from another person was reported. On 30 January 2020, WHO announced that novel coronavirus outbreak is ‘Public Health Emergency of International Concern’ for the world [3].

The epidemic disseminated to other countries and on 12 March WHO announced it as pandemics.

### 1.3.Quarantine beginning in Wuhan

On 23 January 2020, China closed Wuhan city in order to take the pandemic under control. At 02:00 AM Chinese authorities informed Wuhan residents that all the public transport means will be hanged from 10:00 AM. Wuhan residents were not allowed to leave city unless they have permission from authorities. After that, in a few hours in Wuhan, all the travels to and from nearby cities were restricted as well. All cities in Hubei eventually added to this circle^6^6https://www.bbc.com/news/world-asia-china-51217455. 

## 2. Evacuation Acts

Due to the restrictions and pandemic’s course, some countries planned to evacuate their citizens and diplomats from the region. These evacuations firstly done by special flights allowed by Chinese authorities. Japan, USA, Australia, India, France, Germany, and Thailand were taking the first line in evacuating their citizens from the area. Pakistan announced that no citizen will be evacuated from the region due to shortage of treatment facilities in the country in case someone is infected. 

In the first evacuation flight from China to USA that took place on 29 January 2020, 195 people were evacuated. CDC announced that these people will be quarantined in an army base for 72 h and then will be followed up at their residents for 14 days, incubation period of the virus^7^7https://edition.cnn.com/2020/01/30/health/us-coronavirus-thursday/index.html. 

Between 29 January and 6 February, USA brought 808 people back home from Hubei. In departure no passengers showed any symptoms or signs. Quarantine period which was planned as 3 days at the beginning elongated to 14 days. By the 23 February 2020, 28 of these people developed symptoms and 3 of them were tested positive of SARS CoV2. They were transferred to a medical centre later on. The remaining 805 people were fine and 14 days of quarantine period was completed [4].

One of the staff members took role in these evacuation operations claimed that they were not properly educated before flights and there was shortage of equipment. He told that he thought they got the virus already but were not tested when they were back home^8^8https://www.washingtonpost.com/health/2020/02/27/us-workers-without-protective-gear-assisted-coronavirus-evacuees-hhswhistleblower- says/. 

On 29 January, Australia and New Zealand announced that they will collaborate in evacuating their citizens^9^9https://www.nzherald.co.nz/world/news/article.cfm?c_id=2&objectid=12304302. Australian government declared that citizens will be quarantined in Christmas Island for 14 days. Australian Medical Doctors’ Association opposed to the location considering that mentioned island was a place where refugees were put before and many traumas and saddening stories took place there. Australian government evacuated and quarantined 240 citizens including 84 children and 5 babies in Christmas Island on 3 February 2020 despite some opposing ideas in the country^10^10https://www.theguardian.com/world/2020/feb/08/a-hard-and-sad-decision-fleeing-coronavirus-in-wuhan-for-christmas-island. 

Since they learned they will be quarantined in the Christmas Island, problems occurred during evacuation process and some Australians had stressful moments. The staff, who were completely volunteer, were well equipped during this evacuation operation and the airplane was cleaned punctiliously^11^11https://www.abc.net.au/news/2020-02-03/coronavirus-escaping-australians-evacuated-from-wuhan-on-flight/11922638 . It was not thought that it would be necessary that the staff would be quarantined afterwards. Evacuation plane brought tons of medical equipments to China as well^12^12https://www.sbs.com.au/news/after-14-hour-airport-ordeal-australians-board-evacuation-flight-from-wuhan . 

On 5 February, New Zealand government evacuated more than 200 people. One hundred and ninety-three New Zealand citizens and some others from Pacific countries were taken to quarantine base in Whangparao and 35 Australians to the Christmas Island. 

When Australian government stated that there might not be a 3rd evacuation flight, remaining Australian citizens were not satisfied with triage process implemented and thought that they were not valued well by their own government. Luckily, Australian government planned another flight on 8 February and evacuated 266 people including 77 children, 1 baby and 8 students. Passengers were medically checked/examined twice, one before departure and one after landing to Darwin Island. They were quarantined in Darwin Island. Despite the local people’s reaction, government officials told that there would not be any contamination^13^13https://www.abc.net.au/news/2020-02-09/coronavirus-evacuation-flight-arrives-darwin-from-wuhan/11947258?pfmredir=sm&section=world. 

Japan operated its first evacuation flight on 29 January 2020. Among 206 people, 3 were detected positive for coronavirus^14^14https://www.cnbc.com/2020/01/30/japanese-evacuees-from-wuhan-test-positive-for-virus.html. Two out of these 3 were without symptoms. Two Japanese citizens evacuated from Wuhan rejected to be tested for coronavirus^15^15https://www.straitstimes.com/asia/east-asia/evacuation-of-japanese-citizens-from-wuhan-in-spotlight-after-two-returnees-refuse. Japan decided to quarantine people who were tested negative for 2 weeks.

In following days Japan evacuated 210 people on 30 January, 149 people on 31 January, 198 people on 7 February, and 65 people on 17 February. All the passengers were screened for symptoms upon arrival. Portable thermoscanners were used to screen the temperature. In the following, all passengers were interviewed regarding whether they had any symptoms suggestive of upper respiratory tract infection, including fever and cough [5]^16^16https://www.gmanetwork.com/news/news/world/726233/japan-keeps-high-covid-19-alert-as-nationals-return-from-china/story/. 

Singapore evacuated 92 people on 30 January^17^17https://www.thestar.com.my/news/regional/2020/01/30/92-singaporeans-evacuated-from-wuhan-arrive-at-changi-airport. Two of the passengers were tested positive. They did not have any symptoms during the flight but had fever when they entered the country. Some passengers were not also taken to the plane since they had some signs of disease in preflight medical check. On board, passengers put surgical masks on and staff put N95 masks^18^18https://www.straitstimes.com/singapore/some-singaporeans-with-symptoms-of-virus-staying-behind-in-wuhan-even-as-92-areevacuated.

On 9 February, Singapore evacuated another 174 people in second round of evacuation flight. Singapore Ministry of Health declared one of the evacuees diagnosed with COVID-19 on 16 February and on 21 February, another case was confirmed^19^19https://www.channelnewsasia.com/news/singapore/wuhan-virus-coronavirus-second-scoot-flight-hubei-china-12412404. 

On 29 January, South Korea prepared 2 airplanes each having 20 doctors, nurses, and other staff in order to evacuate 700 citizens from Wuhan^20^20https://en.yna.co.kr/view/AEN20200129008351325. They evacuated 368 people on 31 January, 333 people on 1 February, and 140 people on 11 February. Government isolated people for 2 weeks in Asan and Jincheon that were located 80 km south to the capital Seoul. Isolation took place after everyone was medically scanned^21^21http://www.koreaherald.com/view.php?ud=20200212000061,http://www.koreaherald.com/view.php?ud=20200201000057.

On 29 January, Indonesia evacuated its citizens from Wuhan with cabin crew and medical staff^22^22https://jakartaglobe.id/news/plane-to-wuhan-indonesia-starts-evacuation-of-250-citizens-from-coronavirus-epicenter. Out of 245 citizens living in Wuhan, 4 of them refused to go and 3 of them were not allowed by Chinese authorities after being examined. Citizens who got in were taken to Natuna Island^23^23https://www.thejakartapost.com/news/2020/02/02/coronavirus-indonesians-from-wuhan-arrive-in-batam-before-taken-to-natunafor- quarantine.html. Residents in the island protested this decision thinking they would be infected^24^24https://www.nst.com.my/world/world/2020/02/562297/indonesians-protest-use-island-virus-quarantine. On 2 February 2020, another 238 Indonesians were evacuated. After 14 days of quarantine, nobody showing any symptoms, therefore, people got back to their home^25^25https://thediplomat.com/2020/02/after-14-day-quarantine-238-indonesians-from-wuhan-finally-come-home/.

On 31 January, plane carrying 83 English and 41 EU citizens left Wuhan and came to Brize Norton^26^26https://www.bbc.com/news/health-51325192. English passengers were quarantined in Arrowe Park hospital in Wirral. All the passengers were tested before flight and none of them were positive with the test afterwards. EU citizens went to Spain from Brize Norton. In the second evacuation flight on 9 February, total 200 (105 English and other citizens from different countries) were evacuated to England from Wuhan^27^27https://www.bbc.com/news/uk-51430654. 

France organised evacuation flights on 31 January, bringing 180 people; on 3 February, bringing 254 people (65 French and rest of them are from different countries); on 10 February, bringing 35 people; and on 21 February, bringing 64 people. Everyone was quarantined in Normandy^28^28https://www.france24.com/en/20200203-36-evacuated-from-china-to-france-show-virus-symptoms-minister. 

On 1 February, Bangladesh evacuated 316 people from Wuhan, most of them being students. After arrival, these citizens remained in a specifically designed place near Dakka airport for 14 days^29^29https://www.dhakatribune.com/bangladesh/2020/02/01/biman-flight-leaves-wuhan-for-bangladesh.

On 1 February, India evacuated 324 people with Air India flight^30^30https://www.thehindu.com/news/national/324-indians-leave-coronvirus-hit-wuhan-in-air-india-flight/article30707329.ece. In second evacuation flight, 323 Indian and 7 Maldivian were evacuated. Staff took proper measures. Indians were quarantined in 2 different quarantine facilities^31^31https://www.hindustantimes.com/india-news/india-airlifts-second-batch-of-323-citizens-from-china-s-coronavirus-hit-wuhan/ story-s1Zq2SDohOJnIAvegIASQJ.html. In contrast, Pakistan refused to evacuate its students^32^32https://thediplomat.com/2020/02/if-pakistan-wont-evacuate-its-citizens-from-wuhan-india-should/. On 27 February, Indian Air Forces evacuated 76 Indian and 36 foreigners from Wuhan, in total 112 people. This was 3rd evacuation plane sent by India. This 3rd plane also brought 15 tons of medical aid to China^33^33https://economictimes.indiatimes.com/news/politics-and-nation/coronavirus-india-brings-back-36-foreigners-and-76-nationalsfrom- wuhan/articleshow/74328899.cms.

On 1 February, Myanmar evacuated 59 of 63 students from Wuhan airport. Two people were not allowed since they had fever. In arrival all the passengers were quarantined in a block separated for them in Myanmar Army Complex^34^34https://www.bangkokpost.com/world/1847909/myanmar-turns-back-guangzhou-flight.

On 1 February, Mongolia evacuated 31 of its 33 citizens from Wuhan and put them in quarantine after arriving to Ulan Batur, in National Infectious Diseases Centre. Also, on 1 February, Jordan evacuated 71 and Morocco evacuated 167 citizens.

Thailand evacuated 64 people on 1 February and 138 on 4 February under supervision of medical staff^35^35https://www.reuters.com/article/us-china-health-thailand/thailand-evacuates-138-from-virus-hit-wuhan-idUSKBN1ZY0ZF. Six people from these evacuated were hospitalized due to high fever later on and rest of them were quarantined in Sattahip base. On 8 February, it was announced that one of the citizens evacuated has positive test result. 

On 31 January, Germany evacuated 102 Germans and 26 people from other nationalities. Passengers transferred to Germersheim and quarantined for 14 days. During the flight 10 passengers put in isolation. Six of them had symptoms and 2 of them had history of a close contact with people who have coronavirus. Other 2 travelled with isolation including family members. These 10 people were transferred to Frankfurt University Hospital. All PCR results of them came out negative. Remaining 116 were examined in Frankfurt Airport and 1 was transferred to the university hospital since he had fever but his test came out negative as well. One of 115 people under quarantine did not allow to be tested and from other 114, 2 people had positive PCR results [6]. Afterwards these 2 were transferred to hospital and isolated there^36^36https://www.dw.com/en/coronavirus-german-evacuation-flight-from-china-carried-two-infected-people/a-52229955.

On 4 February, 144 people (128 Russians and 16 from other countries) were evacuated by Russia with 2 military airplanes^37^37https://www.bbc.com/russian/news-51387954. Passengers were welcomed by fully equipped medical staff at the airport. They were taken to Siberia for the quarantine. On 5 February, another 147 Russian were evacuated and put in quarantine in Tumen rehabilitation centre^38^38https://www.themoscowtimes.com/2020/02/05/russians-start-2-week-coronavirus-quarantine-after-return-from-wuhan-a69155.

On 4 February, it was announced that 1000 Vietnam citizens will be evacuated and quarantined^39^39https://www.reuters.com/article/us-china-health-vietnam/vietnam-to-quarantine-950-people-returning-from-china-at-military-camps-idUSKBN1ZY0M8. On 10 February, 30 Vietnamese were evacuated from Wuhan. Plane that took off to Wuhan had 11 Chinese citizens going to Wuhan as well. In return it came back to Vietnam with citizens of Vietnam and these people put in quarantine in Dong Ang region after medical scanning^40^40https://vietnamnews.vn/society/592055/30-vietnamese-citizens-return-from-wuhan-safely-isolated.html. The plane was disinfected and shut down for 24 h^41^41https://www.bloomberg.com/news/articles/2020-02-10/vietnam-evacuates-vietnamese-from-china-quarantines-returnees.

On February 1, Turkey performed first evacuation operation in this pandemic by bringing 32 Turkish citizens as well as 6 Azerbaijani, 3 Georgian, and 1 Albanian back to home. Evacuation took place under the leadership of an experienced infectious diseases professor and with a well-educated and equipped medical team. Among the people who are on board, medical team and pilots were in quarantine when they were back home as well. Everyone underwent detailed medical examination before and after flight and no one showed any symptoms. Protective measures were taken for droplet and aerosolization type transmissions. In Ankara after return, nasopharyngeal swab samples were taken for PCR testing in 3rd, 10th, and 14th days. All results were negative. 14 days of quarantine was completed. Medical team and pilots completed last 4 days at their homes. 

Saudi Arabia suspended all Umrah visits on 27 February 2020. They announced first case of COVID-19 on 2.03.2020^42^42https://tr.euronews.com (accessed: 8.04.2020)), (https//www.aa.com.tr (accessed: 8.04.2020).

On March 2020, 21,000 Umrah worshippers returned back to Turkey. People who returned to Turkey (6448 people) after 15.03.2020, were quarantined in 7 different cities and among them, 224 positive cases were reported and taken to treatment. Quarantine period still goes on for some of the mentioned worshippers. Other than Umrah worshippers, 4821 people were brought back home from various countries like Italy, Spain, Germany, Luxembourg, Tunisia, Lebanon, Azerbaijan, Ukraine, and Kazakhstan and put in quarantine in empty student dormitories^43^43https://www.sgkrehberi.com/haber/252990/illere-gore-karantina-sayisi-aciklandi-1-sirada-o-il-var.html (accessed: 8.04.2020) https://www.haberturk.com/kayseri-haberleri/76481125-goruntulu-karantina-altindaki-umreci-saglik-durumumuz-cok-iyi (accessed: 8.04.2020).

In addition, Turkey operated other evacuations from Iraq and Iran too. Lastly on 25 March 2020, 3358 students living in Europe were evacuated from different European countries and put in quarantine in different cities in specially prepared government facilities. This great evacuation process is going on and viral positivity, virus dissemination as well as psychosocial outcomes are being evaluated.

On 7 February 2020, Canada evacuated 174 Canadians. Also, the same day American evacuation plane brought more than 60 Canadians to Vancouver^44^44https://bc.ctvnews.ca/plane-evacuating-canadians-out-of-wuhan-departs-for-vancouver-1.4799867?cache=yes. Passengers were examined by Canadian Public Health Institution teams. They were in quarantine till 21 February, and no one had any symptom or positive test result. On 11 February, Canada evacuated 130 Canadians and 56 family members from Wuhan in their second round of evacuation. These people came to Trenton and entered in quarantine 4545https://globalnews.ca/news/6530909/coronavirus-quarantine-flight-crew-released/.

Malaysia evacuated 120 citizens on 1 February, 107 on 4 February, and 66 on 26 February 4646https://www.channelnewsasia.com/news/asia/malaysia-evacuate-wuhan-coronavirus-arrive-klia-quarantine-12388318 https://www.channelnewsasia.com/news/asia/covid19-air-asia-flight-wuhan-evacuation-arrives-malaysia-12471422

On 2 February, Saudi Arabia evacuated 10 students. On 7 February, Brazil evacuated 34 Brazilians, 4 Polishes, 1 Chinese, and 1 Indian from Wuhan^47^47https://www.reuters.com/article/us-health-coronavirus-evacuation-factbox/factbox-countries-evacuating-nationals-fromcoronavirus- hit-areas-idUSKBN20S0ZU.

On 2 February, Algeria evacuated 36 Algerians, 19 Tunisians, and some Libyan students from the region^48^48https://www.middleeastmonitor.com/20200203-coronavirus-algeria-evacuates-tunisian-nationals-and-libyan-students-from-chinaswuhan/.

On 3 February, Italy evacuated 56 people^49^49https://abcnews.go.com/Health/wireStory/italians-poles-quarantined-coming-back-china-68719781. On 4 February, Iran announced 56 Iranians as well as 59 Iraqis, 24 Syrians, and 1 Lebanese were evacuated from Wuhan. After evacuation, all these 4 countries put their citizens in quarantine for 14 days^50^50https://abcnews.go.com/Health/wireStory/italians-poles-quarantined-coming-back-china-68719781.

Taiwan evacuated 247 people on 3 February, 19 on 21 February, and 361 on 11 March. On the third flight 22 buses and 10 ambulances were ready in airport to bring people to hospitals immediately in case anyone develops symptoms^51^51https://www.taipeitimes.com/News/taiwan/archives/2020/03/12/2003732546 https://focustaiwan.tw/cross-strait/202002050027.

On 9 February, 30 Philippine citizens were evacuated from Wuhan. They were in quarantine in New Clark city in the following 14 days. On 16 February, Nepal evacuated 175 people from Wuhan. Six people could not get in the flight due to medical conditions and 4 citizens decided to stay in Wuhan^52^52https://www.reuters.com/article/us-health-coronavirus-evacuation-factbox/factbox-countries-evacuating-nationals-fromcoronavirus- hit-areas-idUSKBN20S0ZU.

On 19 February, Ukraine evacuated 48 Ukrainians and 29 foreign citizens from Wuhan. Four people meant to come were not allowed by Chinese authorities due to medical inconvenience. All the evacuees were taken to Kharkiv airport and then transferred to National Guard Hospital for 14 days of quarantine. Some Ukrainians attacked the transfer bus to protest decision, tried to block the bus^53^53https://abcnews.go.com/International/hysteria-coronavirus-sparks-violent-protests-ukraine/story?id=69124337.

On 27 February, Colombian Air Forces evacuated 15 Colombians from Wuhan. Passengers put in quarantine. On 4 March, UAE evacuated 215 people from Wuhan. This flight was including 58 Yamani students while 120 Yamani students did not want to come and stayed in China^54^54https://gulfnews.com/uae/health/coronavirus-215-people-evacuated-from-china-to-the-uae-1.70151106. On 4–5 March, Hong Kong evacuated 533 citizens from China with 4 different flights^55^55https://www.reuters.com/article/us-health-coronavirus-hongkong-carrie-la/hong-kong-to-evacuate-stranded-residents-fromchinas- hubei-idUSKBN20Q069.

On 13 March, South Africa evacuated 122 people. South African government decided a quarantine period of 21 days for these people. Several African countries managed to evacuate their citizens from China while some others avoided to do because of insufficient medical experience and facilities at home^56^56https://qz.com/africa/1811001/coronavirus-south-africa-kenya-ghana-unsure-on-student-return/.

People from many countries who live in Wuhan requested to leave and get back to their country and they left so. At the same time some Chinese people who are abroad as tourists meanwhile were stuck outside of China since Wuhan was under a lockdown. Chinese government brought them back home with special flights, being 111 people from Japan on 31 January, 124 people from Malaysia on 31 January as well^57^57https://www.nst.com.my/world/world/2020/01/561321/china-sends-flights-bring-back-hubei-residents-malaysia-thailand. On 31 January and 1 February, total 165 people were brought back from Thailand. On 8 February, 61 people from Indonesia were taken to China^58^58https://time.com/5802348/china-blocking-flights-italy-iran-south-korea-coronavirus/. 

Similar to evacuations from China, some countries evacuated their citizens from new epicentres of the pandemic. China evacuated 800 citizens from Iran with flights on 4, 5, and 11 March. India evacuated 345 people from Iran on 10, 15, and 16 March and put them in quarantine afterwards^59^59https://www.indiatoday.in/india/story/indian-air-force-to-fly-c-17-globemaster-to-evacuate-indians-from-coronavirus-affectediran- 1654005-2020-03-09 https://www.hindustantimes.com/india-news/covid-19-234-indians-stranded-in-coronavirus-hit-iran-arrive-in-india/story- HePm2t2E3DYh2fZe1DfgIM.html.

South Korea evacuated 80 people from Iran on 19 March^60^60http://world.kbs.co.kr/service/news_view.htm?lang=e&Seq_Code=152133.

India evacuated 300 people from Italy on 11 and 15 March^61^61https://www.indiatoday.in/india/story/coronavirus-83-evacuated-from-italy-put-in-army-s-manesar-quarantinefacility- 1654529-2020-03-11 https://www.hindustantimes.com/india-news/218-indians-from-coronavirus-hit-italy-land-in-delhi-will-be-quarantined-for-14-days/ story-vYY3hbhcVC6F8wCW8kScGN.html

They were also put in quarantine when they were back home. On 13 March, Thailand evacuated 89 people from Iran. Israel operated 2 flights to evacuate Israeli students found in Italy on 23 March^62^62https://www.timesofisrael.com/330-israelis-flee-milan-on-rescue-flight-amid-devastating-virus-outbreak/.

### 2.1. Diamond Princess cruise ship

Following a passenger got off from Diamond Princess in Hong Kong tested positive, cruise was quarantined in Japan’s Yokohama City on 3 February with everyone inside.

 Passengers inside the cruise asked their governments to evacuate them due to inappropriate conditions and risks they are facing. Many countries took their citizens from the cruise and put them in quarantine in their homelands.

On 16–17 February, USA government evacuated 329 USA citizens. By 23 February, 36 of these evacuees were tested positive for SARS Cov-2 and following this they were taken under medical supervision. Rest of them were put in quarantine^63^63https://www.who.int/docs/default-source/coronaviruse/who-china-joint-mission-on-covid-19-final-report.pdf.

Australian government evacuated about 180 people from Diamond Princess on 20 February and quarantined them in Darwin. When tested following the arrival, 10 people tested positive for COVID-19 and these people were sent to hospitals^64^64https://www.abc.net.au/news/2020-02-20/cruise-ship-australians-arrive-darwin-for-coronavirus-quarantine/11981394. Others who were not evacuated with this flight were not allowed to enter Australia until 4 March. They were told to complete 14 days of quarantine period before entry. They were also reminded that they may be isolated in accordance with Biosecurity Law 2015^65^65http://theconversation.com/explainer-what-are-the-laws-mandating-self-isolation-and-how-will-they-be-enforced-133757.

South Korea evacuated 7 people from the cruise on 19 February^66^66http://world.kbs.co.kr/service/news_view.htm?lang=e&Seq_Code=151422. Hong Kong evacuated 195 people in total on 20, 21, and 23 February 6767https://www.scmp.com/news/hong-kong/health-environment/article/3064818/about-100-hongkongers-evacuated-diamondprincess. On 21 February, Canada evacuated 200 people^68^68https://www.japantimes.co.jp/news/2020/02/18/national/science-health/canada-diamond-princess-covid19/#.XoH7e4gzbIU. Taiwan evacuated 19 people on 1 February. UK evacuated 32 people on 22 February and India evacuated 124 people on 27 February. All the countries took passengers to the quarantine^69^69https://www.gov.uk/government/news/diamond-princess-evacuation-flight-lands-in-the-uk-foreign-secretarys-statement. Evacuation activities of the countries, names of the countries, the number of the evacuated citizens, the dates of the evacuation, quarantine duration, quarantine places are given in the Table.

**Table 1:** Evacuation acts of countries.

Country	Departured country	Number of evacuees	Date ofevacuation (2020)	Quarantineplace	Quarantineduration
USA	China	195	29 January	March Air Reserve Base (California)	72 h (self isolationup to 14 days)
	China	613	2–6 February	4 U.S. Military Base	14 days
Japan	China	206/210/149/198/65	29 January /30 January /31 January/7 February /17 February	Tokyo	14 days
Singapore	China	92/174	30 January/9 February	Changi	14 days
United Kingdom	China	83 English and41 EU citizens/200	31 January/9 February	Arrowe Park (Wirral)	14 days
South Korea	China	368/333/140	31 January/1 February/11 February	Asan and Jincheon (Seoul)	14 days
	Iran	80	19 March		14 days
Germany	China	128	31 January	Germersheim	14 days
France	China	180/254/35/64	31 January/3 February/10 February/21 February	Normandy	14 days
Turkey	China	32 (+10 other nations)	1 February	Quarantine hospital in Ankara	14 days
	Italy, Spain, Germany, Tunusia, Azerbaijan, Lebanon, Ukraine	4821	15March	Student dormitories in İstanbul, Bolu, Bartın, Tokat	14 days
	Saudia Arabia (Umrah worshipper)	6448	15 March	Student dormitories in Ankara, Eskişehir, Isparta, Kayseri, Konya, Kastamonu, and Bartın	14 days
	EU Countries (Bologna Exchange Students)	3358	23 March	Student dormitories in various cities	14 days
Bangladesh	China	316	1 February	Dakka	14 days
India	China	324/330/112	1 February/9 February/27 February	Delhi and Haryana’s Manesar	14 days
	Iran	345	10, 15, and 16 March	Indian Army Wellness Facility Center	14 days
	Italy	300	11 and 15 March	Indian Army Wellness Facility Center	14 days
Myanmar	China	59	1 February	Myanmar Army Complex	14 days
Mongolia	China	31	1 February	Ulan Bator	14 days	Jordan	China	71	1 February		14 days
Morocco	China	167	1 February		14 days
Thailand	China	64/138	1 February/4 February	Sattahip base	14 days
Australia	China	243/266	3 February/8 February	Christmas Island/Darwin Island	14 days
Indonesia	China	238	2 February	Natuna Island	14 days
Russia	China	144/147	4 February/5 February	Siberia Tyumen	14 days
New Zealand	China	More than 200 people	5 February	Whangparao(35 Australians to Christmas Island)	14 days
Canada	China	174 more than60 (in USA plane)/186	7 February/7 February/11 Febuary	Trenton	14 days
Malaysia	China	120/107/66	1 February/ 4 February/26 February		14 days
Saudi Arabia	China	10	2 February		14 days
Algeria	China	More than 50	2 February		14 days
Italy	China	56	3 February		14 days
Taiwan	China	247/19/361	3 February/21 February/11 March		14 days
Iran	China	140	4 February		14 days
Brazil	China	40	7 February		14 days
Philippines	China	30	9 February	New Clark	14 days
Vietnam	China	30	10 February	Dong Ang region	14 days
Nepal	China	175	16 February		14 days
Ukraine	China	77	19 February	National Guard Hospital	14 days
Colombia	China	15	27 February		14 days
United Arab Emirates	China	215	4 March		14 days
Hong Kong	China	533	4–5 March		14 days
South Africa	China	122	13 March		21 days
Thailand	Iran	89	13 March		14 days
Israel	Italy	330	23 March		14 days
China	Japan	111	31 January		14 days
	Malaysia	124	31 January		14 days
	Thailand	165	1 February		14 days
	Indonesia	61	8 February		14 days
	Iran	800	4, 5, and 11 March		14 days
Taiwan	*D.P. ship	19	1 February		14 days
USA	*D.P. ship	329	16 February		14 days
Australia	*D.P. ship	180	20 February	Darwin Island	14 days
South Korea	*D.P. ship	7	19 February		14 days
Hong Kong	*D.P. ship	195	20, 21 and 23 February		14 days
Canada	*D.P. ship	200	21 February		14 days
UK	*D.P. ship	32	22 February		14 days
India	*D.P. ship	124	27 February		14 days

## 3. Conclusion

Evacuation processes carried some medical risks and social problems. First of all, by evacuating people virus could be transferred from a place to another. In all countries who evacuated their citizens, evacuees were put in quarantine for the following 14 days. Social and psychological traumas have been an important issue to deal with during this extraordinary time. Some evacuees tested COVID-19 positive with PCR testing.

On the other hand, in some countries local residents did not want people in their cities and this caused another issues/problems.

Quarantine process have been implemented in all countries but the way governments implemented it varied.

All evacuees were put in quarantine strictly for 14 days regardless of their social, mental, and economical status. Places prepared for quarantine were different from one country to another.

In addition to health concerns, economic and social anxieties appeared by time and this caused even more problems and made the situation harder.

It is necessary to do more evaluations for different potential difficulties at this point. These include economical, psychological, and social aspects of the evacuation and quarantine practices.

## Acknowledgments

One of the authors Prof Dr. İrfan Şencan was among the members of the evacuation team of Ministry of Health of Turkey, who went to Wuhan to evacuate Turkish citizens.
